# Pharmacogenomics and Epigenetic Regulation Transforming Pediatric Precision Therapeutics

**DOI:** 10.3390/jpm16060329

**Published:** 2026-06-19

**Authors:** Shakta Mani Satyam, Sainath Prabhakar, Tanya Densil, Husham Taha Mohammed, Rashmi Kumari, Mohamed El-Tanani, Abdul Rehman, Ahmad Kharoufeh, Mohammed Dalbah, Mohamed Talat Zaky Mahmoud Eltrabishi

**Affiliations:** 1 Department of Pharmacology, RAK College of Medical Sciences, RAK Medical and Health Sciences University, Ras Al Khaimah 11172, United Arab Emirates; 2 Department of Perfusion Technology, Manipal College of Health Professions, Manipal Academy of Higher Education, Manipal 576104, India; 3 A. J. Institute of Hospital Management, A. J. Hospital and Research Centre, Mangalore 575004, India; 4 RAK College of Pharmacy, RAK Medical and Health Sciences University, Ras Al Khaimah 11172, United Arab Emirates; 5 Department of Pathological Sciences, College of Medicine, Ajman University, Ajman P.O. Box 346, United Arab Emirates

**Keywords:** pediatric pharmacogenomics, precision medicine, developmental pharmacology, epigenetics, drug metabolism, artificial intelligence in healthcare, pharmacokinetics, personalized therapeutics, health equity, universal health coverage

## Abstract

Pediatric drug therapy remains fundamentally challenged by profound interindividual variability driven by dynamic development, genetic, and environmental factors. Although dosing strategies based on age, body weight, or body surface area remain important starting points in pediatric pharmacotherapy, they may not fully capture ontogeny-dependent variability in drug disposition and response. Consequently, clinically relevant differences in efficacy and toxicity may still occur among children receiving similar weight-adjusted doses. Pharmacogenomics offers a promising framework for individualized therapy; however, its clinical translation in pediatrics is limited by developmental variability in gene expression and enzyme activity. Emerging evidence highlights the pivotal role of epigenetic regulation, including DNA methylation, histone modifications, and microRNAs, in modulating pharmacogenetic expression across developmental stages, thereby reshaping drug response trajectories. Concurrently, advances in artificial intelligence and next-generation sequencing enable integration of multidimensional datasets, facilitating predictive modeling of drug efficacy and toxicity. This narrative review provides a comprehensive synthesis of developmental pharmacology, pharmacogenomics, and epigenetic mechanisms, while critically evaluating current translational gaps and implementation challenges. Importantly, it proposes an integrative precision framework that incorporates genetic, epigenetic, and computational insights to optimize pediatric pharmacotherapy. By bridging mechanistic biology with emerging digital health technologies, this work advances a paradigm shift from empirical prescribing toward predictive, adaptive, and individualized therapeutic strategies. The proposed approach holds significant potential to enhance clinical outcomes, minimize adverse effects, and accelerate the realization of precision medicine in pediatric populations.

## 1. Introduction

Precision medicine has emerged as one of the most transformative paradigms in modern healthcare. Within this evolving landscape, pharmacogenomics has gained considerable attention as a pivotal strategy for tailoring drug therapy according to individual genetic characteristics [1,2,3,4]. By identifying genetic determinants that influence drug absorption, distribution, metabolism, and pharmacodynamic responses, pharmacogenomics offers the possibility of optimizing therapeutic efficacy while minimizing adverse drug reactions. Although this approach has gained increasing traction in adult medicine, its application in pediatric populations remains comparatively underdeveloped despite the substantial clinical need.

Children constitute a dynamic and extremely diverse group of patients in a permanent state of physiological change. The dynamic nature of growth and development through infancy, childhood and adolescence can alter the pharmacokinetic and pharmacodynamic characteristics of drugs [5,6]. Changes in hepatic enzyme activity, renal clearance, drug absorption, and receptor binding can result in considerable inter-individual variability in drug response, which cannot be adequately accounted for by weight or age alone [7]. The actual dosing regimens, which are based on weight or body surface area, remain useful initial dosing approaches but may not fully account for the complexity of drug disposition and response in children, potentially contributing to drug toxicity or suboptimal therapeutic efficacy [8]. One of the solutions to the problem of drug overdose may lie in the relatively new discipline of pharmacogenomics [9]. Studies have identified that genetic polymorphisms can significantly affect individual responses to drug therapy [10,11,12]. These polymorphisms can be in genes coding for drug metabolizing enzymes, drug transporters, or drug targets. For example, genetic polymorphisms in the genes coding for the cytochrome P450 enzymes CYP2D6 and CYP2C19 have been shown to affect the metabolism of a wide variety of drugs, including narcotics, antidepressants and proton pump inhibitors [13,14,15]. Genetic polymorphisms affecting drug transporters and immune recognition receptors have also been shown to affect drug disposition and have been implicated in drug hypersensitivity reactions [16]. Because of the rapidly changing physiology of growing children and the individuality of genetic background, pediatrics is a field where pharmacogenomics may have a significant impact on drug response. The clinical consequences of genetic variants differ substantially between children and adults because developmental maturation influences drug absorption, distribution, metabolism, and elimination. Furthermore, the functional impact of genetic variants may change throughout childhood as physiological systems mature, resulting in age-dependent variability in drug response [5,17,18].

**Figure 1:**
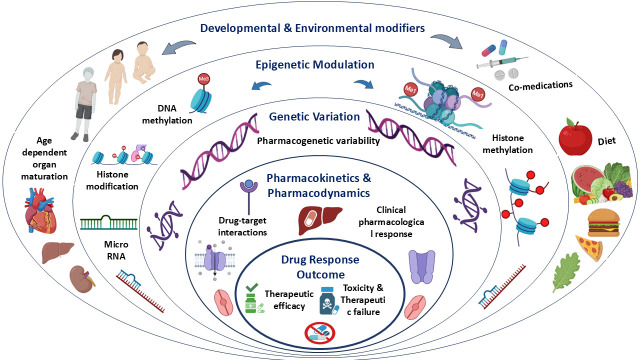
Schematic illustration of determinants influencing drug response variability.

Compared to the impact of genetic polymorphisms on drug response in children, the role of epigenetics is still evolving; the field of drug response and disposition, seen from an epigenetic perspective, is a growing research area. DNA methylation, histone modification, and microRNA expression represent major epigenetic mechanisms regulating gene expression. These processes contribute to developmental changes in pharmacologically relevant genes throughout childhood. These regulatory mechanisms influence gene expression without altering the underlying DNA sequence [19,20,21]. Early life represents a critical period of epigenomic programming during which tissue-specific gene expression patterns are established. Although some epigenetic marks may remain relatively stable, many exhibit dynamic regulation throughout life in response to developmental processes and environmental influences. These modifications may be altered by factors such as diet, infection, disease states, and pharmacological exposures, thereby influencing the expression of pharmacologically relevant genes and contributing to variability in drug disposition and response [22,23]. The convergence of pharmacogenomics and epigenetics represents an emerging frontier in pediatric precision medicine [24,25]. Understanding how inherited genetic variation interacts with developmental epigenetic regulation could significantly enhance the ability to predict drug responses in children. Such insights may help explain interindividual variability that cannot be accounted for by genotype alone and may facilitate the development of more accurate predictive models for individualized therapy (Figure 1).

Figure 1 summarizes the complex interactions among genetic variation, epigenetic regulation, developmental physiology, environmental influences, and pharmacokinetic/pharmacodynamic processes that collectively determine drug response outcomes in children.

Genetic variation constitutes the core pharmacogenetic basis, where polymorphisms in drug-metabolizing enzymes and transporters drive inter-individual variability. Longitudinal pediatric cohort studies have demonstrated substantial developmental changes in drug-metabolizing enzyme expression, transporter activity, and pharmacogenomic phenotypes across infancy, childhood, and adolescence [26,27,28]. Epigenetic modulation includes dynamic regulatory mechanisms such as DNA methylation, histone modifications, and microRNA-mediated control of pharmacogenetic expression. Developmental and environmental modifiers encompass age-related physiological changes, diet, disease states, and drug–drug interactions, collectively shaping variability in drug response across the lifespan.

Another transformative development in this field is the rapid advancement of artificial intelligence and machine learning technologies. Modern healthcare systems generate vast amounts of clinical, genomic, and pharmacological data. AI-driven analytical approaches offer the capacity to integrate multidimensional clinical, genomic, and pharmacological datasets and identify complex patterns associated with drug response. However, most existing models require further validation in pediatric populations before routine clinical implementation [29]. In pediatric pharmacotherapy, where developmental trajectories and genetic variability intersect, such computational tools may prove particularly valuable for generating individualized dosing recommendations and predicting adverse drug reactions.

Despite substantial advances in developmental pharmacology and pharmacogenomics, many proposed precision medicine approaches remain insufficiently validated in pediatric populations. Longitudinal pediatric studies incorporating genomic, epigenomic, developmental, and clinical outcome data are particularly needed to establish the predictive utility, reproducibility, and clinical applicability of emerging precision therapeutic models. Unlike previous reviews that have largely examined pharmacogenomics, epigenetics, or computational modeling separately, this narrative review integrates these complementary domains within a unified pediatric precision medicine framework, highlighting their collective potential to improve individualized therapeutic decision-making in children. This narrative review synthesizes current advances in pediatric precision therapeutics, with a focus on the interplay among developmental physiology, pharmacogenomics, epigenetic regulation, and emerging computational approaches that influence drug response in children. The review examines the genetic and epigenetic determinants of pediatric pharmacotherapy, evaluates established and emerging clinical applications of pharmacogenomics, and explores the potential of artificial intelligence-driven predictive models to support individualized treatment strategies. Furthermore, it highlights key challenges, knowledge gaps, and future directions for translating precision medicine into routine pediatric practice. Collectively, these advances support a transition from empirical dosing paradigms toward data-driven, individualized therapeutic approaches that have the potential to improve treatment efficacy, optimize safety, and enhance clinical outcomes in pediatric populations.

## 2. Literature Search Strategy

A structured narrative literature search was conducted using PubMed and Scopus to identify relevant peer-reviewed English-language publications published between January 2015 and January 2026. This review was conducted as a structured narrative review with the objective of providing a comprehensive thematic synthesis of current evidence in pediatric precision therapeutics. As the focus was on integrating and critically discussing emerging concepts, mechanistic insights, and clinical applications across multiple disciplines, a narrative review methodology was considered more appropriate than a formal systematic review. Therefore, PRISMA-based study selection procedures, quantitative evidence synthesis, and meta-analytic methods were not applied. The search strategy was developed a priori and incorporated both free-text keywords and controlled vocabulary terms, including Medical Subject Headings (MeSH) where applicable. Core search terms included “pharmacogenomics,” “pharmacogenetics,” “pediatric” OR “children,” “developmental pharmacology,” “drug metabolism,” “cytochrome P450,” “CYP2D6,” “CYP2C19,” “pharmacokinetics,” “precision medicine,” “personalized medicine,” “epigenetics,” “DNA methylation,” “histone modification,” “microRNA,” “drug response variability,” “artificial intelligence,” and “machine learning.” To enhance transparency and reproducibility, representative search combinations included: (“pediatric pharmacogenomics” AND “drug metabolism”), (“children” AND “precision medicine” AND “pharmacokinetics”), (“epigenetics” AND “drug response” AND “pediatrics”), (“CYP2D6” OR “CYP2C19”) AND “children,” and (“artificial intelligence” OR “machine learning”) AND “pediatric therapeutics.” The final selection of references was guided by relevance to pediatric precision therapeutics, scientific quality, recency of evidence, and contribution to the thematic objectives of the review. Additional manual screening of reference lists from eligible articles and relevant reviews was performed to identify potentially important studies not captured through electronic database searches. Eligible publications included original research articles, observational studies, clinical trials, pharmacokinetic and pharmacodynamic investigations, pharmacogenomic association studies, translational research studies, systematic reviews, meta-analyses, and narrative reviews that provided substantial relevance to pediatric precision therapeutics. Studies focusing exclusively on adult populations were generally excluded unless they offered important mechanistic, developmental, or translational insights directly applicable to pediatric pharmacology. Three investigators independently screened titles and abstracts, followed by full-text assessment of potentially eligible articles. Literature management and duplicate removal were performed using standard reference management software. Discrepancies regarding study eligibility, relevance, or interpretation were resolved through discussion and consensus among all reviewers. Because this work represents a narrative review rather than a systematic review or meta-analysis, formal inter-reviewer agreement statistics were not calculated; however, consensus-based evaluation was used throughout the screening and selection process to enhance consistency and reduce subjective bias. The selected literature was synthesized thematically to facilitate integration of evidence across key domains, including genetic variability, developmental pharmacokinetics, epigenetic regulation of pharmacogenes, clinical implementation of pharmacogenomics, and emerging artificial intelligence-driven precision medicine approaches. Potential sources of bias were critically evaluated during evidence synthesis, including publication bias, selection bias, language bias, population-specific bias, and methodological heterogeneity among studies. Attention was given to study design, sample size, patient demographics, developmental stage, statistical rigor, reproducibility of findings, and acknowledged study limitations. Areas characterized by limited pediatric evidence, conflicting results, or predominantly preclinical data were interpreted cautiously to avoid overstatement of conclusions. This approach was intended to provide a balanced, clinically relevant, and scientifically rigorous overview of the evolving landscape of pediatric precision pharmacotherapy while acknowledging the current limitations of the available evidence base [30].

## 3. Genetic Foundations of Drug Response in Children

Pharmacogenetic variability arises from multiple forms of genetic variation, including single nucleotide polymorphisms, insertions and deletions, copy number variations, structural variants, and regulatory sequence alterations that collectively influence drug metabolism, transport, efficacy, and toxicity [31]. Genetic variants represent important determinants of interindividual variability in drug response, although environmental, developmental, epigenetic, and disease-related factors also contribute substantially. Although pharmacogenetic information has been derived from adults, it is now recognized that genetic factors significantly affect drug disposition in children, and that the expression of these genetic variations can be affected by development variations in drug disposition and action. Thus, the identification of the genetic basis of drug response is critical to the implementation of personalized medicine in pediatrics [32,33,34].

Although thousands of genes in which polymorphisms are associated with drug response have been identified, the cytochrome P450 (CYP450) genes have garnered the most attention. Cytochrome P450 enzymes are responsible for the metabolism of a substantial proportion of clinically used drugs, highlighting their central role in interindividual variability in drug response [35,36]. Because of their significant role in drug metabolism, there is considerable polymorphism of genes coding for CYP450 enzymes resulting in a wide range of enzymic activity [37]. Among the various genes coding for the CYP450 family, the CYP2D6 gene is a key candidate in drug metabolism studies. This gene has been shown to code for enzymes involved in the metabolism of approximately 20–25% of drugs used in clinical practice [38,39]. CYP2D6 is highly polymorphic and exhibits substantial genetic diversity, including single nucleotide variants, gene deletions, duplications, hybrid genes, and regulatory polymorphisms. Narcotics, psychotherapeutic drugs including tricyclic antidepressants and neuroleptics, as well as certain antiarrhythmic drugs are among those drugs that are CYP2D6 substrates [40,41,42]. More than one hundred CYP2D6* alleles, including coding variants, non-coding regulatory variants, gene deletions, duplications, and hybrid rearrangements, have been identified [43].

CYP2D6 polymorphisms have been studied in children to evaluate their clinical significance [38,44]. The level of expression of hepatic enzymes changes with growth and development, therefore the contribution of any polymorphism will vary with age [32]. These varied expressions are a result of mutations occurring at the coding region of a single gene which results in a decrease in the enzymatic activity of the drug-metabolizing enzymes. Such mutations often result in a reduction in enzymatic activity, which may be further influenced by additional genetic variants, gene deletions, duplications, and gene amplification [38,39]. Gene deletions and duplications modify normal (wild type) gene expression levels. Deletions typically reduce or eliminate gene product production, particularly in homozygous states, whereas duplications increase gene copy number, leading to enhanced gene product expression [45]. In neonates and young infants, poor drug-metabolizing enzyme activity can mask expression of the genotype-related metabolic phenotype. As drug-metabolizing enzymes develop during infancy and childhood, the contribution of genotype to drug response becomes increasingly apparent and may differ substantially from neonatal life [46]. The combined effects of drug-metabolizing enzyme maturation and genotype on the drug response phenotype means that pediatric pharmacogenomics is a dynamic and evolving field. Maturation in combination with genotype is likely to remain an important consideration in the prediction of drug behavior in children. The interaction between prodrugs and their activating catalysts or enzymes is critical to their clinical performance. An excellent example of this is provided by the opioid prodrugs’ codeine and tramadol, and their activation by the CYP2D6 enzyme. Codeine is a prodrug of the opioid morphine, being converted to this active metabolite by O-demethylation catalyzed by CYP2D6. Children with the ultrarapid form of the CYP2D6 gene can metabolize codeine to morphine at a faster rate resulting in elevated morphine concentrations which can lead to respiratory depression and death. There have been reported deaths from overdose with the normally prescribed therapeutic dose of codeine in children with the CYP2D6 gene duplication mutations (genotypes) [47,48]. This has led to regulatory warnings and bans on prescribing codeine to children in several countries. Similarly, children with the poor metabolizer form of the CYP2D6 gene may not obtain sufficient analgesia due to suboptimal production of morphine. Given the risk of life-threatening respiratory depression among CYP2D6 ultrarapid metabolizers, regulatory agencies contraindicate codeine use in children younger than 12 years and recommend extreme caution in older pediatric populations.

In pediatric pharmacogenomics, the gene that is very frequently studied is the CYP2C19 gene. This gene encodes for the enzyme responsible for metabolizing drugs such as some of the proton pump inhibitors, some antidepressants and some antiepileptic drugs [49]. Mutations in the CYP2C19 gene include loss-of-function mutations, including mutations such as CYP2C19 (gene mutation) CYP2C19*2 and CYP2C19*3 and gain-of-function mutations, including mutations such as CYP2C19*17 (gene mutation). Loss-of-function genotypes are associated with increased plasma drug levels. There is decreased drug metabolism. Side effects, such as sedation, may be exaggerated. A gain-of-function genotype is associated with decreased plasma drug levels and hence the drug concentration at its target site may be decreased [50]. Thus, the therapeutic drug effect may be reduced. There is increasing evidence that genetic polymorphisms affecting drug transporters may be another important factor contributing to interindividual variability of drug metabolism. Membrane transport proteins play an important role in the disposition of a wide range of drugs. The drug may be absorbed, distributed and excreted by transport proteins present in various membranes. Clinically relevant CYP2C19 polymorphisms influence proton pump inhibitor exposure and treatment outcomes. Poor metabolizers may experience higher plasma drug concentrations and enhanced acid suppression, whereas ultrarapid metabolizers may exhibit reduced therapeutic response. Genetic polymorphisms affecting drug transporters represent another important source of interindividual variability in drug disposition. Membrane transport proteins regulate the absorption, distribution, and elimination of numerous therapeutic agents. Among these, the hepatic uptake transporter organic anion transporting polypeptide 1B1 (OATP1B1), encoded by the SLCO1B1 gene, plays a critical role in mediating hepatic drug uptake [51,52]. Reduced-function SLCO1B1 variants, particularly the SLCO1B1 c.521T>C allele, decrease transporter activity and can result in elevated systemic drug exposure [53,54,55]. Although statins are the most extensively studied substrates of OATP1B1, several clinically relevant pediatric medications are also affected [56,57,58]. For example, SLCO1B1 polymorphisms have been associated with altered methotrexate pharmacokinetics in children with acute lymphoblastic leukemia, resulting in reduced hepatic uptake, delayed drug clearance, increased systemic exposure, and a higher risk of treatment-related toxicity [59,60,61,62]. Similarly, variability in SLCO1B1 activity has been linked to altered disposition of rifampicin and other antimicrobial agents, potentially influencing therapeutic response and toxicity [63,64,65]. Emerging evidence also suggests a role for SLCO1B1 variants in the pharmacokinetics of selected anticancer agents, including irinotecan and its metabolites [66,67,68,69,70]. These findings highlight the growing importance of drug transporter pharmacogenomics in pediatric precision medicine and demonstrate that transporter-related genetic variation may contribute substantially to variability in treatment outcomes beyond the effects of drug-metabolizing enzymes alone.

Genetic variation in immune recognition pathways is another dimension of pediatric pharmacogenetics. The human leukocyte antigen (HLA) proteins in the immune system display peptides or fragments of foreign substances so that the immune system can identify foreign invaders. A few HLA alleles have been strongly associated with drug hypersensitivity reactions (DHRs) that can sometimes be severe, such as severe cutaneous adverse reactions (SCARs) seen in particular antiepileptic drugs [6,71]. Although these types of adverse drug reactions are rare, it could be useful to know the genotype of a patient before giving them a drug to which they are at high risk to avoid exposure in the unlikely event that they experience a severe reaction. Considering numerous recent pharmacogenomic observations in children, pediatric pharmacogenetics is a highly complex area of research, which cannot be restricted to drug metabolism. Numerous processes may affect the pharmacokinetics and pharmacodynamics of drugs in children: absorption, tissue distribution, interaction with target receptors, immune response and signal transduction. These genes may interact with the numerous other elements of pediatric development, as well as environmental, clinical and therapeutic factors affecting drug effects [6]. As genomics becomes a more integral part of modern health care, pharmacogenetics is bound to play a prominent role in achieving optimal drug effects and safety in children. This is another excellent reason to continue to research drug effects and genes in children.

## 4. Epigenetic Regulation of Drug Response During Pediatric Development

Pharmacogenetic variation is caused by inherited genetic variation. However, inherited genetic variation within gene regulatory regions does not always translate directly into gene expression, and additional layers of gene regulation may be required for the manifestation of pharmacological phenotypes and drug response [72]. Heritable and reversible changes in gene expression that occur without alterations in the underlying DNA sequence are termed epigenetic modifications. Examples of epigenetic regulation include DNA methylation, histone modification, chromatin remodeling, and microRNA-mediated post-transcriptional gene regulation [73]. Increasing evidence indicates that these mechanisms contribute directly to the developmental regulation of pharmacogenes [74,75,76]. For example, age-dependent DNA methylation changes have been associated with the ontogeny of hepatic drug-metabolizing enzymes such as CYP3A4 and CYP2E1, where progressive promoter demethylation correlates with increased gene expression during childhood [74,77]. Similarly, microRNAs including miR-27b and miR-148a have been shown to regulate the expression of nuclear receptors, drug-metabolizing enzymes, and transport proteins involved in pharmacokinetic pathways [78,79]. Histone modifications have also been implicated in developmental changes in CYP450 enzyme expression, influencing metabolic capacity across different stages of pediatric development [80,81]. These observations suggest that drug response in children is determined not only by inherited genetic variation but also by dynamic epigenetic mechanisms that modulate pharmacogene expression throughout growth and maturation. Epigenetic regulation of gene expression can directly influence the activity of drug-metabolizing enzymes, transporters, and drug target genes, making it a crucial determinant of drug response, particularly during key developmental windows [82,83]. The fetal and early postnatal period is a critical window for epigenetic regulation of gene expression in various tissues. This time period is critical for the rapid growth and development of organs, which requires tightly regulated and dynamic gene expression programs for proper organ function and for the neonate to adapt to the postnatal environment [84]. Elucidation of drug action and disposition in developmental stages has identified the role of the epigenome in maturation of drug-metabolizing proteins. Many drugs metabolizing cytochrome P450 enzymes undergo developmental epigenetic changes that affect their expression in children [85].

The functional consequences of DNA methylation depend strongly on genomic context. Methylation within promoter regions is commonly associated with transcriptional repression, whereas methylation within gene bodies or distal regulatory elements may exert different effects. Although developmental DNA methylation may contribute to age-dependent variation in pharmacogene expression, reduced neonatal enzymatic activity is multifactorial and reflects developmental immaturity of transcriptional networks, hormonal regulation, organ maturation, and environmental influences. DNA methylation of cytosines within CpG sequences is one of the most studied mechanisms of gene regulation via an epigenetic mechanism. While DNA methylation at the distance from the transcription start site (TSS) is generally repressive in post-embryonic cells by blocking transcription factors binding to their cognate sequences in gene promoters, promoter regions of drug-metabolizing enzymes undergo increased DNA methylation in early development to switch them off [86]. This DNA methylation is then in a sequential manner, demethylated to switch on and to increase the expression of drug-metabolizing enzymes as the child grows, a phenomenon which is also observed in the well-characterized classical pediatric pharmacokinetic studies in the liver [87]. Recently there have been a few reports on the regulation of genes encoding for cytochrome P450, and in every case, age-dependent demethylation of the promoter region is implicated in the expression of the enzymes at developmental stages. DNA methylation changes occurring during early childhood development explain why neonates and infants have reduced enzymic activity compared with older children and adults, and therefore affect the half-life of drugs, and influence the necessity for weight adjusted, or age-appropriate drug dosing in children [88]. DNA methylation and histone modifications are important regulatory mechanisms of gene expression. Histones are proteins that make up the backbone of chromatin. Modulation of histones by means of enzymatic reactions can influence chromatin architecture and gene expression. In general, the acetylated form (modifications of the protein) has an activating role in gene expression by favoring the relaxed chromatin conformation necessary for gene expression, while deacetylation and certain types of methylation have silencing roles. The enzymes responsible for these modifications, such as histone acetyltransferases (HATs) and histone deacetylases (HDACs), are involved in complex regulatory circuits that can be activated or inactivated in response to various physiological and environmental signals [89,90].

MicroRNAs, which are a class of small non-coding RNAs that regulate gene expression by binding to the complementary sequences in messenger RNA transcripts and resulting in their translational inhibition or degradation, have been found to play a role in drug metabolism in recent years. Several microRNAs directly regulate pharmacogenes involved in drug metabolism and transport, including CYP450 enzymes, ATP-binding cassette transporters, and solute carrier proteins. Through these mechanisms, microRNAs may influence drug exposure, therapeutic efficacy, and susceptibility to adverse drug reactions. Some microRNAs have been found to regulate genes closely related to pharmacogenetics [91]. For example, miR-27b regulates the expression of nuclear receptors of drug-metabolizing enzymes, and miR-148a regulates the expression of genes in liver drug metabolism and transport. However, the epigenetic state is not just a sum of developmental regulatory processes, but also significantly determined by environmental factors such as nutrition, infections, inflammation or drug exposures, which can modulate the epigenetic status and gene expression [92]. Inflammatory cytokines have been shown to suppress CYP450 expression through transcriptional and post-transcriptional regulatory mechanisms. Although epigenetic processes may contribute to these effects, direct causal links between inflammation-induced epigenetic modifications and altered pharmacogene expression remain incompletely characterized in many pediatric settings [93].

Nutritional status and early-life environmental conditions may also influence epigenetic regulation of metabolic pathways. Maternal nutrition during pregnancy, for example, can affect fetal epigenomic programming, potentially altering gene expression patterns relevant to drug metabolism later in life. These findings suggest that pediatric pharmacogenetic phenotypes may be shaped not only by inherited genetic variants but also by early environmental influences that modify epigenetic landscapes. The dynamic nature of epigenetic regulation implies that pharmacogenetic phenotypes in children may change over time. A child carrying a functional allele of a drug-metabolizing enzyme may nevertheless exhibit reduced metabolic activity if epigenetic repression limits gene expression during early developmental stages [94]. Conversely, epigenetic activation during adolescence could amplify the functional consequences of certain genetic variants, leading to increased metabolic capacity. Such temporal variability complicates predictions of drug response based solely on genotype. Recognizing the interplay between genetic polymorphisms and epigenetic regulation is therefore critical for developing accurate models of pediatric pharmacotherapy. Integrating epigenomic data with pharmacogenetic information may help explain variability in drug response that cannot be accounted for by genetic variation alone. Advances in high-throughput sequencing technologies and epigenomic profiling now enable researchers to investigate these interactions in greater detail [95]. Ultimately, understanding how epigenetic mechanisms influence drug metabolism and pharmacological responses during development will enhance the precision of pediatric pharmacotherapy. By incorporating epigenetic insights into pharmacogenomic research, clinicians and scientists may develop more accurate predictive frameworks for individualized drug therapy in children.

## 5. Clinical Applications for Pediatric Pharmacogenomics

### 5.1. Pediatric Oncology

Among all pediatric medical disciplines, oncology represents the most advanced domain for the clinical implementation of pharmacogenomics. In pediatric oncology, pharmacogenomic-guided treatment modifications must be carefully integrated within established disease-specific treatment protocols and multidisciplinary clinical decision-making frameworks. The treatment of childhood cancer is performed generally with chemotherapeutic drugs having a narrow therapeutic index and high toxicity. Since most anticancer drugs display variability in their pharmacokinetic and pharmacodynamic effects among patients, the application of pharmacogenomics for the treatment of children having cancer is highly beneficial, obtaining a maximum drug response while minimizing the adverse effects [9]. Acute lymphoblastic leukemia (ALL) is the most common malignant neoplasm of childhood and is a prime example of pharmacogenomics. Thiopurine drugs such as mercaptopurine (6-MP) are commonly used in the maintenance phase of therapy for ALL [96]. The enzyme thiopurine methyltransferase (TPMT) inactivates 6-MP by methylating it to methylated 6-MP derivatives. Genetic polymorphism affecting the expression of TPMT is well characterized. Some alleles encode less active TPMT enzyme molecules that are associated with low TPMT activity. It has been observed that leukemia patients with low TPMT activity achieve higher concentrations of the more toxic thioguanine nucleotides in their haemopoietic cells. Thus, these patients are at a higher risk of experiencing severe hematologic toxicity (leukopenia, anemia and thrombocytopenia) that in turn may lead to severe infections threatening life. Genotype-guided thiopurine dose adjustment based on thiopurine methyltransferase (TPMT) genotype has become a standard practice in the treatment of children with cancer [97]. Children who are heterozygous for mutations in the TPMT gene are on reduced dose of thiopurines and those who are homozygous deficient for TPMT require substitution with alternative drugs and/or significantly reduced dose of thiopurines [98]. Genotype-guided thiopurine therapy has led to a substantial reduction in the adverse drug effects in children, while not compromising the anti-tumor activity of these drugs. Genotype-guided thiopurine therapy is therefore considered as one of the pioneering examples of precision pharmacology in children [99]. In addition to TPMT, NUDT15 has emerged as a clinically important pharmacogene, particularly among Asian and Hispanic populations. Reduced NUDT15 activity is strongly associated with thiopurine-related myelosuppression and is now incorporated into several pharmacogenomic dosing recommendations for thiopurine therapy [100,101]. Pharmacogenomics of thiopurines, drugs widely used in the treatment of chronic inflammatory diseases such as Crohn’s disease, ulcerative colitis and rheumatoid arthritis, have known side effects that can limit their effectiveness. Another critical pharmacogenetic determinant of thiopurine toxicity involves the NUDT15 gene, which encodes a nucleotide diphosphatase involved in the detoxification of thiopurine metabolites. Loss-of-function variants in NUDT15 have been strongly associated with severe thiopurine-induced leukopenia, particularly in Asian and Hispanic populations [102,103]. Identification of these variants has led to refined dosing recommendations and further improvements in treatment safety. Although thiopurines, drugs used in the treatment of several forms of childhood cancer, have been the object of several pharmacogenetic studies, few studies have examined other drugs used in the treatment of pediatric oncology. However, there are genetic variations in the DPYD gene that are associated with the fluoropyrimidines (such as 5-fluorouracil and capecitabine) metabolism and can cause severe gastrointestinal and hematological side effects [104]. Some genetic variations in folate pathway genes (such as MTHFR and SLC19A1) have also been correlated with methotrexate pharmacokinetics and side effects [105]. Finally, genetic variations in drug transporters (such as ABCB1 and SLCO1B1) can influence intracellular drug concentrations and thereby affect both therapeutic efficacy and toxicity [106,107]. Pediatric oncology is rapidly moving toward the integration of both germline pharmacogenomics (PGx) and somatic tumor genomics into routine therapeutic decision-making [108,109]. Germline pharmacogenomics provides information regarding inherited variants that influence drug absorption, distribution, metabolism, transport, and toxicity, thereby helping clinicians individualize drug selection and dosing. Examples include TPMT and NUDT15 variants that predict thiopurine-related myelosuppression and SLCO1B1 variants associated with altered methotrexate disposition and toxicity [100,110,111]. In contrast, somatic tumor genomics characterizes acquired genetic alterations within malignant cells that drive tumor behavior, treatment responsiveness, and resistance mechanisms [112,113]. Identification of tumor-specific molecular abnormalities can facilitate risk stratification, guide targeted therapy selection, and support treatment adaptation based on tumor biology. The integration of germline and somatic genomic information therefore provides a more comprehensive precision medicine framework in which treatment decisions are informed not only by the child’s capacity to metabolize and tolerate anticancer agents but also by the molecular characteristics of the tumor itself. Such an approach has the potential to maximize therapeutic efficacy, minimize treatment-related toxicity, improve risk-benefit assessment, and ultimately enhance clinical outcomes in pediatric cancer patients. As genomic technologies become increasingly accessible, combined germline–somatic profiling is expected to play an increasingly important role in the development of individualized treatment strategies and next-generation precision oncology programs for children [108,114,115].

### 5.2. Neuropsychiatric Pharmacogenetics

Pharmacotherapy for pediatric neuropsychiatric disorders presents substantial challenges due to wide interindividual variability in drug response and the complexity of neurodevelopmental processes. Medications such as selective serotonin reuptake inhibitors (SSRIs), tricyclic antidepressants, stimulants, and antipsychotic agents are frequently prescribed to treat conditions including depression, anxiety disorders, attention-deficit hyperactivity disorder, and autism spectrum disorders. However, therapeutic outcomes vary widely among children, and adverse effects such as sedation, behavioral disturbances, or metabolic complications may occur. Pharmacogenetic variation in cytochrome P450 enzymes plays a major role in determining plasma concentrations of many neuropsychiatric medications. CYP2D6 and CYP2C19 polymorphisms significantly influence metabolism of commonly prescribed antidepressants and antipsychotics [116]. Children with poor metabolizer phenotypes may experience elevated plasma drug levels, increasing the risk of adverse effects such as excessive sedation or extrapyramidal symptoms. Conversely, ultrarapid metabolizers may clear medications rapidly, resulting in subtherapeutic drug concentrations and inadequate symptom control [116,117]. In addition to pharmacokinetic variation, pharmacodynamic factors also contribute to variability in treatment outcomes. Genetic polymorphisms in neurotransmitter transporter genes, including the serotonin transporter gene SLC6A4, may influence antidepressant response by altering synaptic serotonin signaling [118]. Similarly, variants in dopamine receptor genes may affect responsiveness to stimulant medications used in attention-deficit hyperactivity disorder. Environmental influences and developmental neurobiology further complicate prediction of treatment response. Brain maturation, hormonal changes during adolescence, and psychosocial stressors all interact with pharmacogenetic factors to shape therapeutic outcomes. Consequently, personalized treatment strategies in pediatric psychiatry increasingly emphasize integrated pharmacogenomic approaches that consider both genetic and environmental determinants of drug response [119].

### 5.3. Pharmacogenomics in Infectious Diseases

Host pharmacogenetic variation may influence antimicrobial pharmacokinetics, treatment response, and susceptibility to drug-related toxicity in children. However, antimicrobial resistance primarily arises from genetic and adaptive mechanisms within microbial pathogens rather than host pharmacogenomic variation. An example is the metabolism of isoniazid (INH), a first-line drug used to treat tuberculosis. Isoniazid exerts antimicrobial activity following activation by the mycobacterial catalase-peroxidase enzyme KatG. In humans, NAT2 polymorphisms influence isoniazid acetylation and clearance, thereby affecting systemic drug exposure and risk of toxicity. Genotypic variation leads to individuals being classified as slow, intermediate or rapid acetylators. Slow acetylators develop higher concentrations of INH in the blood and are at a greater risk of developing drug-induced hepatotoxicity and peripheral neuropathy. Rapid acetylators may require a higher dose of INH to achieve therapeutic concentrations [120,121]. Glucose-6-phosphate dehydrogenase deficiency is another important factor. This is an inborn error of metabolism that affects red blood cells. The enzyme deficiency leads to accumulation of metabolites that are toxic to the red blood cells. G6PD deficiency represents an important determinant of susceptibility to drug-induced hemolytic anemia following exposure to oxidant medications. G6PD deficiency is a pharmacogenomic factor and children with G6PD deficiency are at risk of developing hemolytic anemia when given oxidant drugs such as some of the antimalarial drugs. Hence, screening is recommended in endemic areas before giving the drugs [122]. Genetic variation may influence the pharmacokinetics of drugs given to children with HIV infection who are on antiretroviral therapy. Several polymorphisms were identified in the CYP2B6 gene which were associated with the metabolism of efavirenz, and which may explain the large inter-patient variability in plasma drug concentrations and related side effects [123]. Pharmacogenetic insights can guide the administration of antimicrobials in children, enabling safer and more effective therapy tailored to individual genetic profiles.

**Figure 2:**
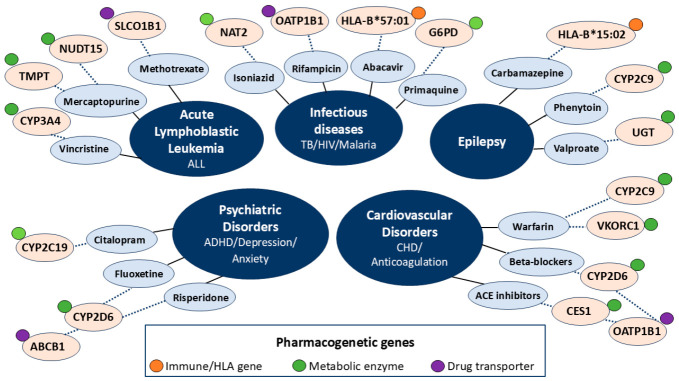
Integrated pharmacogenetic network in pediatric medicine.

Key pharmacogenetic interactions influencing drug metabolism and therapeutic response in pediatric patients are summarized in Figure 2 and Table 1.

In Figure 2, the schematic depicts an integrated pharmacogenetic network linking major pediatric disease categories, commonly prescribed drugs, and key pharmacogenes. Central disease nodes (dark blue) represent primary therapeutic areas, including acute lymphoblastic leukemia, infectious diseases (tuberculosis, HIV, malaria), epilepsy, psychiatric disorders (ADHD, depression, anxiety), and cardiovascular disorders (congenital heart disease and anticoagulation). Surrounding drug nodes (light blue) indicate commonly used medications, such as mercaptopurine and methotrexate in oncology; isoniazid, rifampicin, and abacavir in infectious diseases; and warfarin and beta-blockers in cardiovascular conditions. Pharmacogenetic gene nodes (pink) are further classified into functional groups: metabolic enzymes (green), including cytochrome P450 isoforms (e.g., CYP2D6, CYP2C19, CYP2C9) and phase II enzymes (TPMT, NUDT15, NAT2); drug transporters (purple), such as SLCO1B1 and ABCB1; and immune-related genes (orange), including HLA-B57:01 and HLA-B15:02, associated with severe drug hypersensitivity reactions. Solid lines represent established therapeutic links between drugs and disease states, whereas dashed lines indicate pharmacogenetic influences on drug metabolism, efficacy, or toxicity.

Importantly, the strength of evidence supporting pharmacogenomic implementation varies substantially across different gene–drug pairs. Variants in TPMT, NUDT15, CYP2D6, and CYP2C19 are supported by robust clinical evidence and international prescribing guidelines, making them among the most clinically actionable pharmacogenomic markers currently available. In contrast, many emerging pharmacogenomic associations identified through candidate gene studies or genome-wide analyses remain exploratory and require independent validation before routine clinical implementation. Careful distinction between validated and investigational biomarkers is essential to avoid overestimation of predictive utility and to ensure responsible integration of pharmacogenomics into pediatric clinical practice.

## 6. Implementation Science and Regulatory Frameworks

Despite mounting evidence that pharmacogenomic-guided therapy may result in better health outcomes, pharmacogenomics has yet to be widely adopted in standard pediatric care [139,140]. Implementation science, an emerging field of research, addresses the need to understand how genomic knowledge can translate into practice and improvement in patient health. Several international organizations, including research networks and regulatory agencies, have started to produce guidelines based on the currently available evidence which clinicians can use to implement pharmacogenomics in their practice. For example, the Clinical Pharmacogenetics Implementation Consortium (CPIC) has produced genotype-based dosing guidelines for several genes/drugs that are also relevant to children. The Dutch Pharmacogenetics Working Group (DPWG) is actively developing clinical guidelines to integrate pharmacogenetic information into prescribing decisions, helping clinicians determine when and how a drug should be used based on a patient’s genetic profile [141]. More information is being provided in drug labeling in the form of Pharmacogenomic (PGx) markers based on findings made during clinical trials. More drugs are being granted FDA and EMA approval with PGx biomarkers being highlighted in the drug label to alert prescribers of known genetic variations that may lead to a difference in drug efficacy, increased risk of adverse drug reactions or require adjustment in dose [142].

However, several challenges continue to limit widespread implementation in pediatric medicine. One major barrier is the relative scarcity of pharmacogenomic data derived specifically from pediatric populations. Many pharmacogenetic associations were initially discovered in adult cohorts, and extrapolating these findings to children may not always be appropriate due to developmental differences in drug metabolism [140,143]. Another challenge involves disparities in genomic representation across populations. Most pharmacogenomic research has been conducted in populations of European ancestry, potentially limiting the generalizability of findings to diverse global populations [139,144]. Expanding genomic studies to include underrepresented populations will therefore be essential for achieving equitable precision medicine. Infrastructure limitations also present practical barriers. Integration of genetic test results into electronic health records, development of clinical decision support tools, and clinician education regarding pharmacogenomics are necessary for effective implementation. Healthcare systems must also address issues related to cost, accessibility, and ethical considerations surrounding genomic testing in children. Successful implementation of pediatric pharmacogenomics requires integration across laboratory medicine, clinical pharmacology, informatics, and healthcare delivery systems. Beyond generating genetic information, implementation frameworks must address clinician education, standardized interpretation of results, integration into electronic health records, clinical decision support, reimbursement pathways, and long-term outcome evaluation.

The identification of reliable surrogate biomarkers represents an important objective in pediatric precision therapeutics. Biomarkers derived from pharmacogenomic, epigenomic, transcriptomic, proteomic, and metabolomic platforms may facilitate early prediction of drug efficacy, toxicity, and treatment response while reducing dependence on invasive procedures or prolonged clinical observation. Such biomarkers may be particularly valuable in neonates and young children, where direct assessment of therapeutic outcomes can be challenging.

The implementation of precision medicine in pediatric populations also raises important ethical considerations. Children represent a vulnerable population requiring additional safeguards during clinical research and genomic testing. Issues including informed parental consent, assent from older children, data privacy, long-term storage of genomic information, return of incidental findings, and equitable access to advanced diagnostics require careful consideration. Ethical frameworks that balance scientific advancement with patient protection will be essential for responsible implementation of pediatric precision medicine.

## 7. Artificial Intelligence and Predictive Modeling on Pediatric Pharmacotherapy

Recent advances in computational biology and artificial intelligence (AI) have created new opportunities to integrate complex biomedical datasets for personalized medicine. Several computational approaches contribute to pediatric precision therapeutics, each serving distinct purposes [29]. Machine-learning algorithms identify complex patterns within large clinical and genomic datasets to generate predictive models of treatment response [145,146]. Physiologically based pharmacokinetic (PBPK) models use mechanistic representations of organ physiology, developmental biology, and drug characteristics to simulate drug disposition across pediatric age groups. Population pharmacokinetic models quantify variability in drug exposure across patient populations and support individualized dose optimization [147,148,149]. Together, these complementary approaches provide increasingly sophisticated tools for pediatric drug development and precision prescribing.

**Figure 3:**
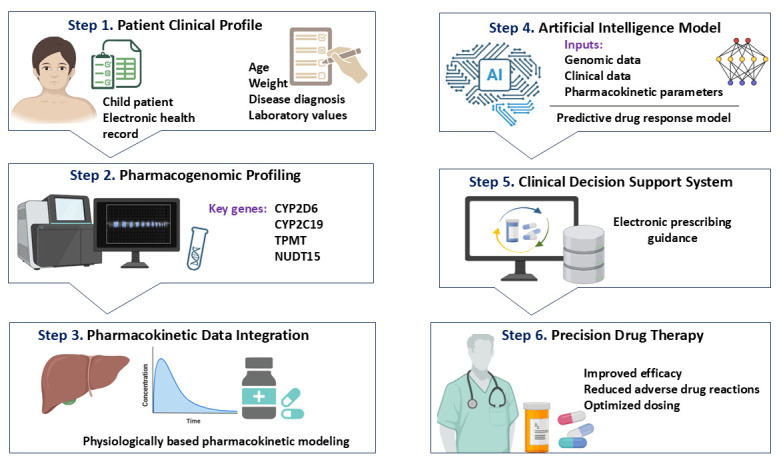
Schematic representation of the integration of pharmacogenomics and artificial intelligence for precision drug therapy.

Artificial intelligence (AI) is increasingly being applied across multiple domains of pediatric precision therapeutics to improve individualized treatment decisions and optimize clinical outcomes. In dose optimization, AI-driven Bayesian forecasting models have been successfully employed to personalize vancomycin dosing by integrating patient-specific pharmacokinetic parameters and therapeutic drug monitoring data [150,151]. In pediatric oncology, machine-learning algorithms are being developed to predict methotrexate toxicity, enabling early identification of high-risk patients and facilitating individualized treatment adjustments [152,153]. Within pediatric intensive care settings, AI-based predictive models are being explored to support sepsis management by optimizing antimicrobial selection, treatment timing, and clinical decision-making [154,155]. Similarly, in neonatal medicine, advanced pharmacokinetic prediction platforms incorporating AI and machine-learning approaches are being utilized to account for the unique physiological characteristics of newborns, thereby improving drug dosing accuracy and therapeutic safety [156,157]. Collectively, these applications demonstrate the growing potential of AI to enhance precision medicine approaches in pediatric pharmacotherapy through more accurate prediction, monitoring, and individualized therapeutic interventions. Particularly in pediatric oncology, therapeutic decisions remain embedded within highly standardized disease-specific treatment protocols for conditions such as acute lymphoblastic leukemia, Hodgkin lymphoma, and rhabdomyosarcoma. Consequently, pharmacogenomic or artificial intelligence-derived recommendations should complement rather than replace multidisciplinary oncologic decision-making. Any modification of established treatment regimens must be carefully evaluated within the context of protocol requirements, disease characteristics, and overall clinical benefit–risk assessment. AI-driven models can incorporate genotype data, enzyme ontogeny profiles, organ function parameters, and environmental factors to generate more precise dosing recommendations. Such models may significantly improve therapeutic outcomes in clinical settings where drug response variability is high (Figure 3). Importantly, these computational models require rigorous validation in diverse pediatric populations before routine clinical implementation.

In Figure 3, the schematic illustrates a stepwise translational pipeline integrating clinical, genomic, and pharmacokinetic data to enable individualized drug therapy. The process begins with the acquisition of patient-specific clinical data, including age, weight, disease diagnosis, and laboratory parameters. This is followed by pharmacogenomic profiling using whole-genome sequencing or targeted testing to identify variants in key pharmacogenes (e.g., CYP2D6, CYP2C19, TPMT, and NUDT15) that influence drug metabolism. These data are integrated with physiologically based pharmacokinetic (PBPK) modeling to predict drug disposition and exposure profiles. Subsequently, multimodal inputs are processed through an artificial intelligence–based model to generate individualized drug response predictions. The outputs are incorporated into clinical decision support systems to guide evidence-based prescribing and dose optimization. Collectively, this framework enables precision drug therapy, improving therapeutic efficacy, minimizing adverse drug reactions, and optimizing dosing in pediatric patients.

Beyond individualized dose optimization, artificial intelligence offers the potential to integrate genomic, epigenomic, transcriptomic, metabolomic, pharmacokinetic, clinical, and environmental data into dynamic systems-level models of drug response. Such models may continuously adapt to developmental changes occurring throughout childhood and identify complex interactions between genetic susceptibility, developmental maturation, disease states, and environmental exposures. By capturing these multidimensional relationships, artificial intelligence may improve prediction of therapeutic efficacy, adverse drug reactions, treatment adherence, and long-term clinical outcomes. These advances support a transition from static genotype-guided prescribing toward continuously learning precision medicine systems capable of providing age-specific therapeutic recommendations across the pediatric lifespan.

The successful implementation of pediatric pharmacogenomics requires integration across multiple levels of healthcare delivery. Beyond genetic testing itself, effective clinical translation depends on clinician education, standardized interpretation frameworks, electronic health record integration, clinical decision support systems, reimbursement policies, and equitable access to testing. Prospective implementation studies have demonstrated that pharmacogenomic-guided prescribing can reduce adverse drug reactions and improve therapeutic precision; however, widespread adoption remains limited by infrastructure requirements, cost considerations, limited pediatric-specific evidence, and variability in clinical practice guidelines. Future implementation efforts should focus on generating pediatric outcome data while simultaneously developing sustainable healthcare delivery models that support routine pharmacogenomic testing.

One of the many promises of genomics is that pharmacogenomic information can be integrated into electronic health records (EHRs) and be incorporated into clinical decision support (CDS) tools in clinical practice [158]. These systems can provide real-time alerts when prescribing medications associated with known pharmacogenetic risks. For example, if a child carries a genetic variant affecting metabolism of a particular drug, the system can automatically recommend an alternative medication or adjusted dosage. Deep learning approaches are also being explored to analyze complex interactions among genetic variants, epigenetic markers, and environmental exposures. These models may uncover previously unrecognized determinants of drug response, enabling the development of more comprehensive precision medicine frameworks. However, the successful integration of AI into pediatric pharmacotherapy will require careful validation of predictive models, transparent algorithms, and strong safeguards to protect patient privacy and data security [159]. Despite their potential, deep-learning approaches face several important limitations in pediatric medicine. Model performance may be constrained by relatively small pediatric datasets, class imbalance, missing longitudinal data, and population heterogeneity across developmental stages. Furthermore, many deep-learning models function as “black-box” systems with limited interpretability, which may reduce clinician confidence and complicate regulatory implementation. Addressing these challenges will require transparent model development, multicenter validation studies, and rigorous evaluation of clinical utility before widespread adoption.

## 8. Future Directions in Developmental Pharmacogenomics

The practice of pediatric precision pharmacotherapy in the future will inevitably involve the integration of multiple layers of biological information to generate systems biology models that can be used to predict drug response in the individual child [4]. Advances in high-throughput sequencing technology mean that costs and times for genomic sequencing are falling rapidly and so early-life pharmacogenomic screening for therapeutic use is on the horizon [160]. Pre-emptive pharmacogenomic testing during childhood could provide clinicians with lifelong information regarding drug metabolism and pharmacological susceptibility. Such data could be incorporated into electronic health records and used to guide prescribing decisions throughout a patient’s lifetime [161].

Genomics is a rapidly evolving field of science, but epigenomics, transcriptomics, proteomics and metabolomics are emerging fields with high-throughput analysis of data becoming an increasingly large component [4]. Understanding how developmental processes affect drug response and integrating all these data types will enhance our understanding of drug response and development. It will be important to build an epigenomic map of the life of children and understand how environmental exposure and disease affect gene expression and how they impact drug metabolism. We can generate large real-world datasets for genomic and clinical information. Many population-scale biobanks and genomic studies have been carried out across the world to study the pharmacogenomic variations across different populations. Incorporating these variations in our models not only improves their accuracy but also allows for a universal applicability of precision medicine [162,163].

Ethical considerations remain central to pediatric precision medicine, including informed consent, assent, privacy protection, genomic data security, incidental findings, and equitable access to advanced technologies [164]. Although artificial intelligence and machine learning have demonstrated substantial promise for improving dose prediction, adverse event detection, and therapeutic optimization, relatively few models have yet demonstrated sufficient clinical utility, prospective validation, and safety to support routine therapeutic decision-making in pediatric practice. Consequently, most current applications should be viewed as supportive decision tools requiring further validation rather than autonomous clinical systems. Emerging genomic and computational technologies are rapidly transforming pediatric pharmacotherapy by enabling integration of genetic, clinical, and environmental data to guide personalized treatment decisions. Technological innovations that are accelerating the implementation of precision pharmacotherapy in pediatric medicine are summarized in Table 2.

Artificial intelligence encompasses several distinct methodological approaches. Machine-learning algorithms identify patterns within large datasets and generate predictive models. Physiologically based pharmacokinetic models simulate drug disposition using mechanistic biological parameters, whereas population pharmacokinetic models quantify variability across patient populations. Although these approaches show considerable promise, relatively few pediatric models have yet demonstrated sufficient prospective validation, clinical utility, safety, and cost-effectiveness to support routine therapeutic decision-making. Furthermore, deep-learning models may suffer from limited interpretability, dataset bias, overfitting, and inadequate representation of diverse pediatric populations, highlighting the need for rigorous validation before widespread clinical adoption.

The implementation of pediatric precision medicine raises important ethical considerations related to informed consent, assent, data privacy, genomic data ownership, and equitable access to advanced technologies. These challenges are particularly relevant because children represent a vulnerable population and genetic information may have lifelong implications. In parallel, surrogate biomarkers including circulating microRNAs, epigenetic signatures, inflammatory mediators, and multi-omics profiles are emerging as promising tools for monitoring drug response and toxicity. However, their clinical utility requires prospective validation in diverse pediatric populations before routine implementation.

An equally important consideration is the need to ensure equitable implementation of precision medicine technologies. Significant disparities in genomic database representation, healthcare infrastructure, access to testing, and socioeconomic resources may limit the generalizability and accessibility of emerging precision medicine strategies. Future efforts should prioritize inclusion of diverse pediatric populations to ensure that advances in pharmacogenomics and artificial intelligence benefit children across different ethnic, geographic, and socioeconomic backgrounds.

## 9. Conclusions

This narrative review advances the evolving field of pediatric precision therapeutics by integrating developmental pharmacology, pharmacogenomics, epigenetic regulation, and emerging artificial intelligence-driven approaches into a unified conceptual framework for individualized drug therapy. The evidence synthesized in this review supports the concept that pediatric drug response is shaped by complex interactions among genetic variation, developmental maturation, epigenetic regulation, environmental exposures, and disease-specific factors throughout childhood. A key conclusion of this review is that genotype alone is often insufficient to explain interindividual variability in therapeutic efficacy and toxicity among pediatric patients. Developmentally regulated epigenetic mechanisms, including DNA methylation, histone modifications, and microRNA-mediated gene regulation, may substantially influence the functional expression of pharmacogenes across different stages of growth and development. Consequently, pediatric precision medicine must move beyond static genetic prediction toward integrated models capable of capturing the biological complexity of childhood.

The review further highlights how advances in next-generation sequencing, multi-omics technologies, physiologically based pharmacokinetic modeling, machine learning, and artificial intelligence are creating unprecedented opportunities to integrate genomic, epigenomic, pharmacokinetic, clinical, and environmental data into predictive therapeutic frameworks. Such approaches have the potential to transform pediatric pharmacotherapy from a reactive process based on empirical dose adjustment into a proactive strategy capable of anticipating therapeutic response, toxicity risk, and individualized dosing requirements before treatment initiation. Importantly, this review identifies critical translational challenges that must be addressed before this vision can be fully realized, including limited pediatric-specific evidence, developmental heterogeneity, underrepresentation of diverse populations in genomic datasets, implementation barriers within healthcare systems, and ethical considerations surrounding genomic testing in children. Future progress will require large-scale longitudinal studies, prospective validation of predictive models, integration of pharmacogenomic decision support into clinical workflows, and equitable access to emerging precision medicine technologies. Collectively, the evidence supports a paradigm shift from conventional empirical prescribing toward adaptive precision therapeutics that integrate developmental, molecular, and clinical information across the pediatric lifespan. By bridging mechanistic biology, translational pharmacology, and computational medicine, this framework provides a foundation for the next generation of pediatric precision healthcare and offers a roadmap toward safer, more effective, and truly individualized therapeutic interventions for children worldwide.

## Figures and Tables

**Table 1:** Clinically Relevant Pharmacogenetic Gene–Drug Interactions in Pediatric Medicine.

Gene	Drug/Class	Mechanism	Clinical Impact	Therapeutic Area
CYP2D6 [124,125]	Codeine, Tramadol	Altered pro-drug activation	Toxicity in ultrarapid metabolizers or reduced efficacy in poor metabolizers	Pain management
CYP2C19 [126,127]	SSRIs, Proton pump inhibitors	Altered metabolic clearance	Variable plasma drug levels affecting efficacy and toxicity	Psychiatry, Gastroenterology
TPMT [128,129,130]	Mercaptopurine, Azathioprine	Reduced thiopurine metabolism	Severe myelosuppression risk	Pediatric oncology
NUDT15 [97,131]	Thiopurine drugs	Impaired detoxification of thiopurine metabolites	Leukopenia and treatment toxicity	Pediatric oncology
SLCO1B1 [56,58,132,133,134]	Statins, chemotherapeutic agents	Reduced hepatic drug uptake	Elevated systemic drug concentrations	Cardiology, oncology
NAT2 [120,135,136]	Isoniazid	Variable acetylation rate	Altered toxicity and efficacy	Infectious diseases
CYP2B6 [137,138]	Efavirenz	Altered drug metabolism	Increased neurotoxicity risk	HIV therapy

**Table 2:** Emerging Technologies Enabling Precision Pediatric Pharmacotherapy.

Technology	Description	Application in Pediatrics	Clinical Potential
Next-generation sequencing [139,161]	Rapid genome and exome sequencing	Identification of pharmacogenetic variants	Early genomic screening
Epigenomic profiling [73,89,90,91]	Analysis of DNA methylation and histone modifications	Understanding developmental regulations of drug metabolism	Dynamic prediction of drug response
Artificial intelligence [4,29,165]	Machine learning models integrating clinical and genomic data	Predictive dosing algorithms	Individualized treatment strategies
Real-world genomic databases [31,141]	Population-scale genomic and clinical datasets	Pharmacogenomic association studies	Improved predictive models
Clinical decision support systems [18,136,144,158]	Integration of genomic data into electronic health records	Real-time prescribing guidance	Safer drug prescribing

## Data Availability

All data arising from this study are included within the article.

## Abbreviations

The following abbreviations are used in this manuscript:

Abbreviation	Full Form
PGx	Pharmacogenomics
NGS	Next-Generation Sequencing
AI	Artificial Intelligence
SNP	Single Nucleotide Polymorphism
CYP450	Cytochrome P450
CYP2D6	Cytochrome P450 2D6
CYP2C19	Cytochrome P450 2C19
TPMT	Thiopurine Methyltransferase
NUDT15	Nucleoside Diphosphate-Linked Moiety X-Type Motif 15
SLCO1B1	Solute Carrier Organic Anion Transporter Family Member 1B1
NAT2	N-Acetyltransferase 2
CYP2B6	Cytochrome P450 2B6
G6PD	Glucose-6-Phosphate Dehydrogenase
HIV	Human Immunodeficiency Virus
SSRIs	Selective Serotonin Reuptake Inhibitors
ALL	Acute Lymphoblastic Leukemia
6-MP	6-Mercaptopurine
DPYD	Dihydropyrimidine Dehydrogenase
MTHFR	Methylenetetrahydrofolate Reductase
SLC19A1	Solute Carrier Family 19 Member 1
ABCB1	ATP Binding Cassette Subfamily B Member 1
DHRs	Drug Hypersensitivity Reactions
SCARs	Severe Cutaneous Adverse Reactions
HLA	Human Leukocyte Antigen
MeSH	Medical Subject Headings
TSS	Transcription Start Site
HATs	Histone Acetyltransferases
HDACs	Histone Deacetylases
miR	MicroRNA
EHRs	Electronic Health Records
CDS	Clinical Decision Support
PGx	Pharmacogenomic
FDA	Food and Drug Administration
EMA	European Medicines Agency
